# Acute toxic encephalopathy following bromadiolone intoxication: a case report

**DOI:** 10.1186/s12883-020-02034-2

**Published:** 2021-01-07

**Authors:** Quan Li, Wei Yu, Yun Qu, Jin-Qiu Wang, Ning Mao, Hai Kang

**Affiliations:** 1 Department of Emergency, Affiliated Yantai Yuhuangding Hospital of Qingdao University, 264000 Yantai, China; 2 Department of Neurology, Affiliated Yantai Yuhuangding Hospital of Qingdao University, Yantai, China; 3 Department of Radiology, Affiliated Yantai Yuhuangding Hospital of Qingdao University, Yantai, China

**Keywords:** Bromadiolone, Poisoning, Toxic encephalopathy, Superwarfarin

## Abstract

**Background:**

Clinically, bromadiolone poisoning is characterized by severe bleeding complications in various organs and tissues. Bromadiolone-induced toxic encephalopathy is extremely rare. Here, we report a special case of bromadiolone-induced reversible toxic encephalopathy in a patient who had symmetrical lesions in the deep white matter.

**Case presentation:**

A 23-year-old woman mainly presented with dizziness, fatigue, alalia and unsteady gait after the ingestion of bromadiolone. The laboratory examinations showed normal coagulation levels. Brain magnetic resonance imaging (MRI) showed apparent diffusion restriction in the bilateral deep white matter. The clinical manifestations and MRI alterations were reversible within one month of treatment with vitamin K. The neuropsychological assessment showed no neurodegenerative changes at the 2-year follow-up.

**Conclusion:**

With the increased use of bromadiolone as a rodenticide, more cases of ingestion have been reported annually over the past several years. Bromadiolone-induced toxic encephalopathy has no special clinical manifestations and is potentially reversible with timely treatment. Because of the reversible restricted diffusion on diffusion-weighted images (DWI) and low apparent diffusion coefficient (ADC) values, transient intramyelinic cytotoxic oedema is thought to be the cause rather than persistent ischaemia. The underlying pathophysiological mechanism is still unknown and may be coagulant-independent. This clinical case extends the current knowledge about neurotoxicity in cases of bromadiolone poisoning and indicates that MRI is useful for the early detection of bromadiolone-induced toxic encephalopathy.

## Background

Bromadiolone, known as superwarfarin, is a second-generation dicoumarin rodenticide [1]. With the emergence of warfarin-resistant rats, bromadiolone, as a more potent rodenticide, has been developed and widely used around the world. Unfortunately, with the increased use of bromadiolone, accidental human poisoning has gradually emerged in recent years [1]. Patients who have ingested bromadiolone usually present with unexplained bleeding in various sites; however, acute reversible encephalopathy without coagulopathy following bromadiolone intoxication has rarely been reported. Brain magnetic resonance imaging (MRI) is one of the most useful tools for detecting bromadiolone-induced toxic encephalopathy. We present a case of acute toxic encephalopathy in a patient who attempted suicide by ingesting bromadiolone.

## Case presentation

A 23-year‐old woman was admitted to the emergency department (ED) at Yuhuangding Hospital, Yantai, China on April 1, 2018, because of dysphoria and altered consciousness. Two days before admission, the patient attempted suicide by ingesting 15 g of 0.5% bromadiolone, and she did not initially seek medical help. One day before admission, the patient experienced dizziness, fatigue, alalia, and unsteady gait. She was sent to a community hospital for treatment. She was treated with traditional therapy, including gastric lavage and catharsis (medicine and dosage unknown). Three hours before admission to our hospital, she progressively became more dysphoric with decreased consciousness. She was then sent to our hospital for further treatment. There was no relevant medical history.

On physical exam, her temperature was normal, with a blood pressure of 107/68 mmHg and a pulse of 97 beats/min. Her abdomen was soft, without tenderness on palpation. There were no signs of bleeding in her skin or organs. Neurological examination showed that the patient was profoundly agitated, confused, dysarthric, and ataxic with nystagmus, muscular hypertonia, a soft neck, and negative bilateral Babinski reflex.

The initial laboratory results on April 1, 2018, were as follows: activated partial thromboplastin time (APTT), 29.5 s; prothrombin time (PT), 11.6 s; and international normalized ratio (INR), 0.98. There were no abnormalities in the coagulation indexes, even upon subsequent re-examination. Renal function, liver function, electrolytes, routine bloodwork, and urinalysis were all normal.

**Fig. 1:**
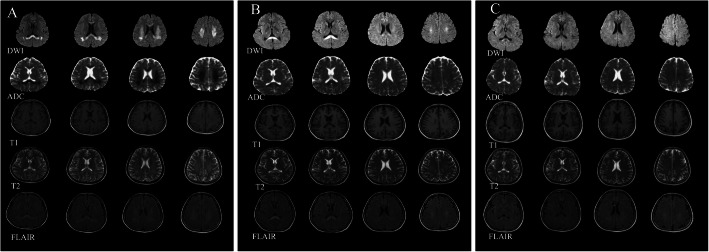
MRI findings during the initial emergency department visit and follow-up. **a** The MRI scan showed lesions in the corona radiata, bilateral centrum semiovale, bilateral posterior limb of the internal capsule and corpus callosum, extending laterally into the posterior deep white matter. **b** A subsequent MRI scan performed 9 days later showed an obvious limitation of the diffusion in the posterior corpus callosum, and there were significant improvements in the other areas that were previously damaged. **c** The MRI scan obtained at the 1-month follow-up visit showed that the abnormal signal alterations were mostly resolved

There was no visible abnormality observed on the patient’s brain computed tomography (CT) scan. The MRI scan of the brain showed the following: slightly long T1 and T2, significantly high signal of fluid-attenuated inversion recovery (FLAIR), hyperintensity of diffusion weighted imaging (DWI), and hypointensity of the apparent diffusion coefficient (ADC) values in the bilateral corona radiata, centrum semiovale, basal ganglia region and corpus callosum region, revealing multiple white matter lesions (Fig. 1a). Considering that the emergence of disordered coagulation indexes may be delayed for a few days after bromadiolone exposure, vitamin K was administered immediately. In this patient, treatment with vitamin K1 was continued over several weeks.

## Outcome and follow‐up

After treatment with vitamin K1, the patient’s neurologic symptoms improved to normal within 5 days. Her PT and INR values were stable between 11 s and 12.3 s and between 0.86 and 1.04, respectively. A subsequent MRI scan performed 9 days later showed an obvious limitation of diffusion in the posterior corpus callosum, and there were significant improvements in the other areas that were previously damaged (Fig. 1b). A follow-up brain MRI 1 month after ED presentation showed that the abnormal signal alterations had almost completely disappeared (Fig. 1c). A psychiatric consultation was arranged for further evaluation and treatment. A neurological physical examination was conducted to detect neurologic deficits during the 2-year follow-up. No neurologic deficits were detected in the patient during the follow-up period. The Mini-Mental State Examination (MMSE) was administered to detect cognitive impairment during the follow-up period. Her average MMSE score was 28, which was within normal limits. She was followed for 2 years and did not show any evidence of neurodegenerative disease.

## Discussion and conclusion

Toxic encephalopathy encompasses a wide spectrum of conditions because of the differences among the various substances. The clinical manifestations and imaging features of toxic encephalopathy may vary based on the different causes of the disorder. Bromadiolone-induced acute toxic encephalopathy is extremely rare, especially without accompanying coagulation disorders.

Bromadiolone is a 4-hydroxycoumarin derivative that shows high potency and long-acting anticoagulation by inhibiting vitamin K epoxide reductase [2]. The most common clinical manifestations of bromadiolone ingestion involve bleeding in various tissues and organs, with the primary cause of death being intracranial haemorrhage [3]. Laboratory examinations commonly show noticeably prolonged APPTs and PTs and increasing INRs in bromadiolone-poisoned patients. It is noteworthy that the primary clinical manifestations may emerge several days after bromadiolone ingestion due to its long half-life, and the exact manifestations can vary extensively.

The associations between haemorrhage and elevated coagulation indexes with superwarfarin exposure has been well documented. This case reported a rare event of bromadiolone-induced, coagulant-independent acute reversible toxic encephalopathy. In this report, the patient’s symptoms included dizziness, fatigue, alalia, dysphoria and unsteady gait. The laboratory examinations showed normal coagulation indexes, including the PT, APTT and INR.

MRI of the brain shows diffusion-restricted lesions in the deep white matter, extending into the corpus callosum. Based on the reversible restricted diffusion on DWIs and ADC maps, transient intramyelinic cytotoxic oedema was considered one of the possible causes.

However, the exact pathophysiological mechanism underlying the development of bromadiolone-induced cytotoxic lesions in the brain remains unclear. Bromadiolone is known as one of the superwarfarins, and it mainly affects vitamin K cycling [4]. Vitamin K is a common cofactor for the enzyme γ-glutamyl carboxylase (GGC), which plays an important role in neuronal and glial cell physiology [5]. In the CNS, deficiency of vitamin K can decrease the carboxylation of protein in the brain, leading to reduced sulfatide synthesis; sulfatide synthesis is critical to the proper formation of the myelin sheath [5]. Warfarin administration has been shown to lead to decreased sulfatide synthesis levels in at least 40% of rodent brains [6]. Thus, the effect of bromadiolone on vitamin K cycling may be one of the pathophysiological mechanisms underlying toxic encephalopathy. In addition, superwarfarins have been known to have vitamin K–independent cytotoxic effects [7, 8]. A recent animal study showed that superwarfarins have direct toxic actions on primary neural cells in the CNS by activating glial inflammation, which ultimately leads to neuronal damage [9]. Glial activation can initiate inflammatory cascade responses and stimulate the release of glutamate, block the reuptake of glutamate, and then increase extracellular glutamate [10–12]. Increasing extracellular glutamate has an excitotoxic effect on aquaporins (AQPs) and sodium-potassium pumps, which promotes water molecule influx into astrocytes and neurons and ultimately leads to cytotoxic oedema [13].

Interestingly, a previous study from M-L Wang et al. also reported bromadiolone-induced CNS lesions with abnormal coagulation indexes [14]. The MRI imaging manifestations in their report were similar to those in ours. The difference was that our patient did not show disordered coagulation function, which indicates that acute toxic encephalopathy following bromadiolone poisoning may be a coagulant-independent process. To our knowledge, this is the second report of acute toxic encephalopathy following bromadiolone poisoning. The lesion regions seemed particularly concentrated in the posterior corpus callosum, which showed more apparent restricted diffusion than other regions on DWIs and ADC maps. It can be speculated that one of the reasons for varying diffusion restriction (cytotoxic oedema) in this condition is that the corpus callosum may have special receptors or protein gradients from the posterior to the anterior. There is some relevant evidence to support this speculation, given that the posterior corpus callosum has a higher density of glutamate receptors and aquaporin 4 (AQP4) [15–17]. This higher density tends to lead to the development of cytotoxic oedema in the posterior, which can progress when lesions occur [18]; however, the exact pathophysiology remains unknown. Although no signs of coagulopathy were identified, the patient was given large and repeated doses of vitamin K. After treatment with vitamin K1, the patient finally recovered without any sequelae. No evidence of ongoing neurodegeneration was discovered at the 2-year longitudinal neuropsychological follow-up visit.

In conclusion, bromadiolone is one of the most widely studied superwarfarins. Ingestion of bromadiolone is typically seen in emotionally disturbed patients. The main clinical manifestation of bromadiolone exposure is haemorrhage because of anticoagulation. Acute bromadiolone-induced encephalopathy is rare and potentially reversible. The presence of coagulopathy may certainly be a clue to the clinical diagnosis, but normal coagulation levels cannot be used to exclude a diagnosis of bromadiolone poisoning because of the long half-life of bromadiolone. The continuous replenishment of vitamin K is an effective treatment. Therefore, it is important for emergency physicians and neurologists to recognize the potentially fatal symptoms of bromadiolone toxicity to achieve a timely diagnosis and early treatment to prevent irreversible damage. The regions of restricted diffusion were located in the deep white matter, mostly concentrated in the posterior corpus callosum. The cause was thought to be intramyelinic cytotoxic oedema; however, the exact pathophysiology is still unknown. This clinical case extends the current knowledge about acute neurotoxicity due to bromadiolone ingestion. Further study is needed to deepen the understanding of the pathogenesis of bromadiolone-induced acute encephalopathy.

## Data Availability

All data and material supporting our findings are contained within the manuscript.

## Abbreviations

CT: Computed tomography
MRI: Magnetic resonance image
DWI: Diffusion-weighted imaging
APTT: Activated partial thromboplastin time
PT: Prothrombin time
INR: International normalized ratio
CNS: Central nervous system
CLOCCs: Cytotoxic lesions of the corpus callosum
(GGC): γ‐glutamyl carboxylase
(ADC): Apparent diffusion coefficient
